# Analysis of HIV Protease Killing Through Caspase 8 Reveals a Novel Interaction Between Caspase 8 and Mitochondria

**DOI:** 10.2174/1874357900701010039

**Published:** 2007-12-27

**Authors:** Alicia Algeciras-Schimnich, Anne-Sophie Belzacq-Casagrande, Gary D Bren, Zilin Nie, Julie A Taylor, Stacey A Rizza, Catherine Brenner, Andrew D Badley

**Affiliations:** 1 Division of Infectious Diseases, Mayo Clinic, Rochester, MN 55905, USA; 2 Program in Translational Immunovirology and Biodefense, Mayo Clinic, Rochester, MN 55905, USA; 3 University of Versailles St-Quentin, UMR CNRS 8159, LGBC, 45 avenue des Etats-Unis, Versailles, France

## Abstract

Human Immunodeficiency Virus (HIV) protease initiates apoptosis of HIV-infected cells by proteolytic cleavage of procaspase 8, creating a novel peptide termed casp8p41. Expression of casp8p41 alone is sufficient to initiate caspase-dependent cell death associated with mitochondrial depolarization. Since casp8p41 does not contain the catalytic cysteine at position 360, the mechanism by which casp8p41 initiates apoptosis is unclear. We demonstrate that casp8p41 directly causes mitochondrial depolarization and release of cytochrome c with downstream caspase 9 activation. Moreover, death induced by casp8p41 requires the presence of mitochondria, and in intact cells, casp8p41 colocalizes with mitochondria. These results illuminate a novel mechanism of cell death induced by a caspase 8 cleavage fragment whereby mitochondrial interaction leads to depolarization and cytochrome c release.

## INTRODUCTION

Since early in the HIV epidemic, it has been recognized that HIV protease is intrinsically cytotoxic, a feature which allowed a cell viability screening approach to identify compounds which ultimately lead to HIV protease inhibitors. Increased understanding of the biologic role of HIV protease has illuminated at least two unpredicted characteristics. First, HIV protease is active within the cytosolic compartment of infected cells [1], and second, the substrate specificity of protease is sufficiently degenerate that a variety of host cell proteins are intracellular substrates for HIV protease [2-6]. For instance, apoptosis mediated by HIV protease is preceded by cleavage of Bcl-2, and based on *in vitro* cleavage, a putative cleavage site of Bcl-2 by HIV protease has been identified [4].

We have recently demonstrated that one such substrate for HIV protease is procaspase 8 and both *in vitro* and *in vivo* HIV protease cleaves procaspase 8 between phenyl alanine 355 and phenyl alanine 356, generating a 41 kd cleavage fragment we call caspase 8 p41 (casp8p41) [1]. Ectopic expression of casp8p41 is sufficient to initiate apoptosis, which is associated with caspase activation, however, the sequence of casp8p41 is notable for missing the active site cysteine at position 360 [1]. Consequently, it remained unknown how casp8p41 causes cell death.

A paradigm for death receptor initiated cell death involves Fas-associated death domain (FADD) clustering at the inner cell membrane following death receptor oligomerization [7]. FADD then recruits procaspase 8 through homophilic interaction of the amino-terminal death effector domains. This complex brings procaspase 8 molecules in close proximity and permits the low constitutive activity present in procaspase 8 zymogens to cleave and activate caspase 8 into the conical heterodimer. In the case of casp8p41, the absence of cysteine 360 makes it unlikely that casp8p41 would activate other procaspase 8 molecules. Therefore, we considered two alternate possible mechanisms of cytotoxicity: 1) acting as a FADD-like scaffold to recruit procaspase 8 and facilitate induced proximity auto-activation of native procaspase 8, or 2) a caspase 8 independent mechanism of cytotoxicity. The purpose of the present study was to determine which of these mechanisms was responsible for the cytotoxicity of casp8p41.

## MATERIAL AND METHODS

### Cell Lines and Transfections

HeLa, Jurkat, and I9.2 cells were purchased from ATCC. Cells were maintained in Dulbecco Modified Eagle Medium (DMEM) or RPMI1640 (GIBCO, Grand Island, NY) supplemented with 10% (v/v) FBS. To transfect HeLa cells for confocal microscopy studies, exponentially growing HeLa cells were seeded on glass coverslips and transfected with pGFP, pGFP-casp8FL, or pGFP-casp8p41 plasmids using Lipofectamine™ transfection reagent (Invitrogen, Carlsbad, CA) following the manufacturer’s protocol. Jurkat and I9.2 T cells were transfected with 10 μg of pGFP control or pGFP-casp8p41 per 10^7^ cells using a square wave electroporator (BTX, San Diego, CA) at 320V for 20 msec. Where indicated, 20 μM ZVAD-fmk, IETD-fmk, or DMSO control were added immediately following transfection.

### Cell Viability Assay

Cell viability was assessed after 8 hours of transfection using a cell titer glow luminescent assay (Promega, Madison, WI), which determines cell viability based upon quantitation of adenine triphosphate (ATP) as a marker of metabolically active cells, according to the manufacturer’s instructions.

### Cell-Free System

Jurkat cell extracts were prepared, as previously described [5]. Briefly, cells (0.5 x 10^6^ cells/ml) were harvested by centrifugation at 1600 rpm for 5 minutes at 4ºC. The cell pellet was washed twice with ice-cold PBS (pH 7.4), followed by a single wash with ice-cold caspase buffer (20 mM PIPES, 100 mM NaCl, 10 mM DTT, 1 mM EDTA, 0.1% CHAPS, 250 mM sucrose, pH 7.2). After centrifugation, the cells were resuspended with two volumes of ice-cold complete caspase buffer, which was supplemented with protease inhibitors (1 mM PMSF, 10 μg/ml leupeptin, 2 μg/ml aprotinin), and then transferred to a 2 ml Dounce homogenizer. After sitting on ice for 15 minutes, the cells were disrupted with 50 strokes using a B-type pestle (Fisher Scientific Ltd, Nepean, ON, Canada). The nuclei were removed by centrifugation at 1000 x g for 10 minutes at 4ºC. To remove the mitochondria, the cytosols were further centrifuged twice at 20,000 x g for 15 minutes at 4ºC. The resultant suspension was incubated with either recombinant GST, GST-casp8p41 or recombinant caspase 8 (positive control) for 2 hours.

For Western blot analysis, 200 μg of cytosolic proteins were fractionated on 12% polyacrylamide gels, and transferred onto PVDF membranes (Millipore, Bedford, MA). The membranes were blotted with primary antibodies as follows: anti-caspase 9 (Medical & Biological Laboratories Co., Watertown, MA), anti-cytochrome c and anti-Hsp70 (Santa Cruz Biotechnology, Santa Cruz, CA). After washing, blots were incubated with HRP linked secondary antibodies and developed using SuperSignal (Pierce, Rockford, IL) following the manufacturer’s protocol.

### Flow Cytometry

Change in mitochondria transmembrane potential as a measure of apoptosis was determined in Jurkat and I9.2 cells transfected by GFP and GFPcasp8p41 after 8 hours of transfection. Briefly, 1 x 10^6^ cells were harvested, and stained with 250 nM TRME (Invitrogen, Carlsbad, CA) at 37ºC for 20 minutes. TMRE staining in the GFP-positive cells was analyzed by flow cytometry using a FACScan (Becton Dickinson Immunocytometry Systems, San Jose, CA). A decrease in TMRE staining was indicative of loss of mitochondrial transmembrane potential. Analysis was performed using CellQuest software (Becton Dickinson, San Jose, CA).

### Mitochondria Staining and Confocal Microscopy

HeLa cells were transfected for 6 hours with GFP, GFP-casp8FL, or GFP-casp8p41 and then stained with 20nM MitoTracker^®^  Red (Invitrogen, Carlsbad, CA) for 45min at 37ºC. After staining, the cells were washed, fixed with 2% paraformaldehide, and coverslips mounted with DAPI containing Vectashield (Vector Laboratories, Burlingame, CA.). Laser-scanning confocal microscopy was performed using a Zeiss LSM-510 (Carl Zeiss Inc., Thornwood, NY).

### Isolation of Mouse Liver Mitochondria and Measurements

Mitochondria were isolated from mouse liver (Balb/c, female, 6-8 weeks) by differential centrifugation and purified on Percoll gradient as described [8]. For swelling and depolarization measurements, the mitochondria were diluted in the hypo-osmotic buffer (10 mM Tris-Mops, pH 7.4, 5 mM succinate, 200 mM sucrose, 1 mM Pi, 10 µM EGTA, 2 µM rotenone). The mitochondrial swelling was measured by the decrease in absorbance at 540 nm at 37°C. The depolarization of the mitochondria was measured by the rhodamine 123 (1 µM) fluorescence dequenching (excitation: 485 nm, emission: 535 nm, Molecular Probes) at 37°C. For cytochrome c and AIF release, mitochondria (40 µg of proteins) were incubated for 30 min at 37°C, and then centrifuged at 6800 g for 10 min at 4°C. Proteins contained in the supernatants were precipitated by methanol:chloroform [4:1, vol], resuspended in Laemmli sample buffer and separated by SDS-PAGE (15%) and transferred to PVDF membranes (Millipore, Hampshire, UK). After overnight incubation at 4°C with either anti-cytochrome c (BD Biosciences, Le Pont de Clay, France) or anti-AIF (Chemicon, Hampshire, UK), proteins were detected by using the ECL method according to the manufacturer's instructions (Amersham Pharmacia Biotech, Rockford, IL). When not indicated, products were from Sigma (St. Louis, MO).

## RESULTS

### Native Procaspase 8 is Not Required for Caspase 8 Cytotoxicity

**Fig. (1):**
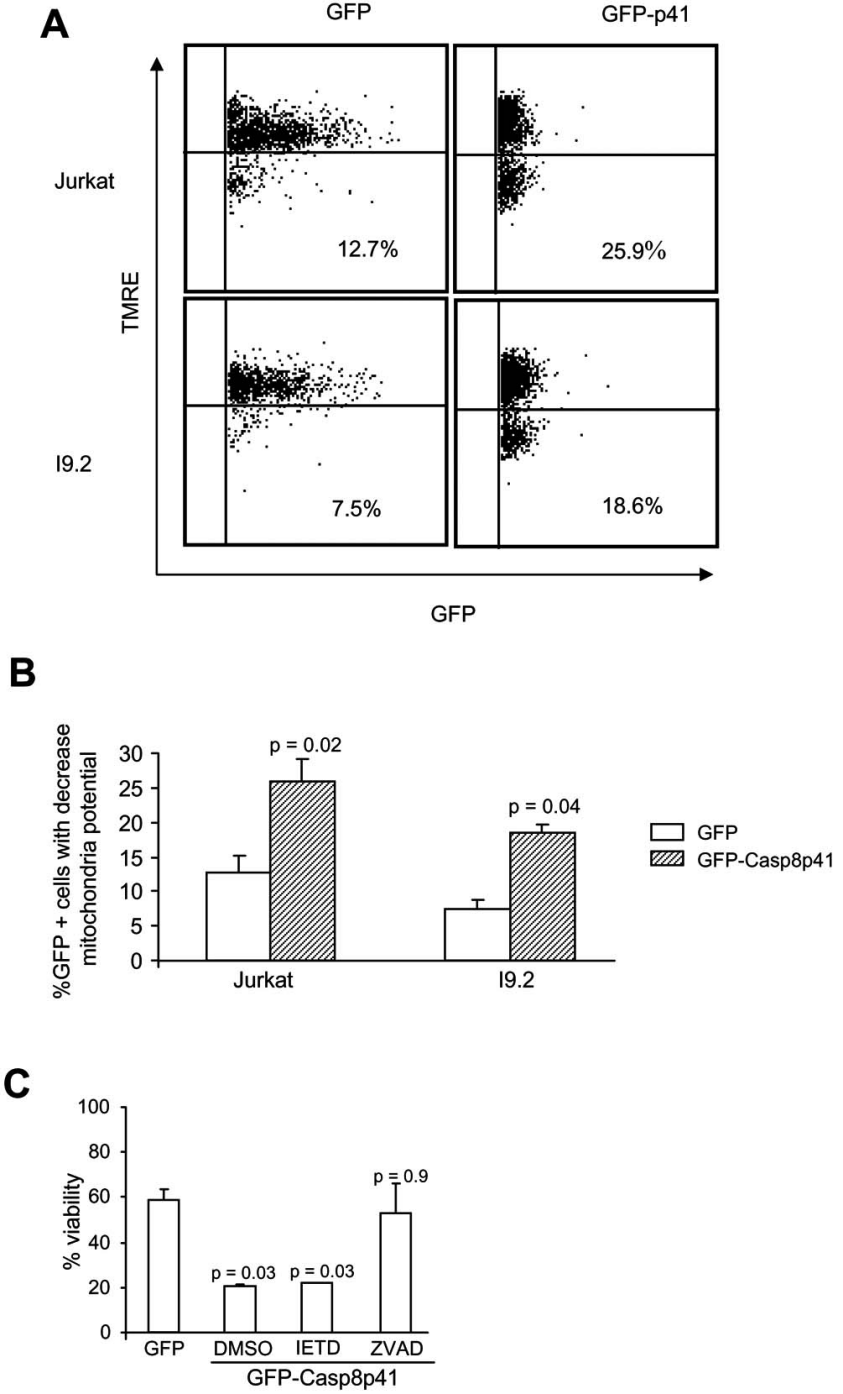
**(A) Casp8p41 induced apoptosis is independent of caspase 8 activity.** Jurkat and I 9.2 cells were transfected with plasmids containing GFP or GFP-casp8p41 for 8 hours. Cells were harvested and mitochondria transmembrane potential measured on the GFP-positive cells by TMRE staining. **(B)** Pool results of three replicates of Fig. **(1A)**. **(C)** Jurkat cells were transfected with plasmids containing GFP or GFP-casp8p41, and immediately after transfection, cells were incubated with the indicated caspase inhibitors for 8 hours. Cells were harvested and cell viability determined using a cell titer glow luminescent assay.

The induced proximity model of casp8p41 cytotoxicity predicts that casp8p41, which contains two DEDs but no catalytic active site, acts as a scaffold to recruit procaspase 8, which leads to auto-activation of native procaspase 8. This model requires that cells contain native procaspase 8, and therefore, we tested whether casp8p41 was cytotoxic to I9.2 cells, which are deficient for procaspase 8. I9.2 cells and the parental Jurkat cell line were transfected with GFP casp8p41 or GFP empty vector control and analyzed for apoptosis within the GFP positive population. As measured by TUNEL staining (data not shown) or loss of mitochondrial transmembrane potential (Fig. **1A**), the proportion of GFP-positive cells that acquired apoptotic features was not different between Jurkat and I9.2 cells, demonstrating that the absence of procaspase 8 is not sufficient to render cells resistant to the apoptotic effects of casp8p41. To confirm this result, we assessed whether caspase 8 inhibitor (Z-IETD Fmk) would alter cell death induced by casp8p41, and whether its effects would differ from those of a pan-caspase inhibitor (Z-VAD-Fmk). As expected, expression of casp8p41 resulted in a significant loss of viability that was not altered by Z-IETD-Fmk, further suggesting that casp8p41-induced death does not require caspase 8 activity (Fig. **1B**). In contrast, the pan-caspase inhibitor, Z-VAD-Fmk, significantly inhibited death induced by casp8p41, confirming the caspase dependence of death induced by casp8p41.

### Death Induced by casp8p41 Requires Mitochondria

**Fig. (2):**
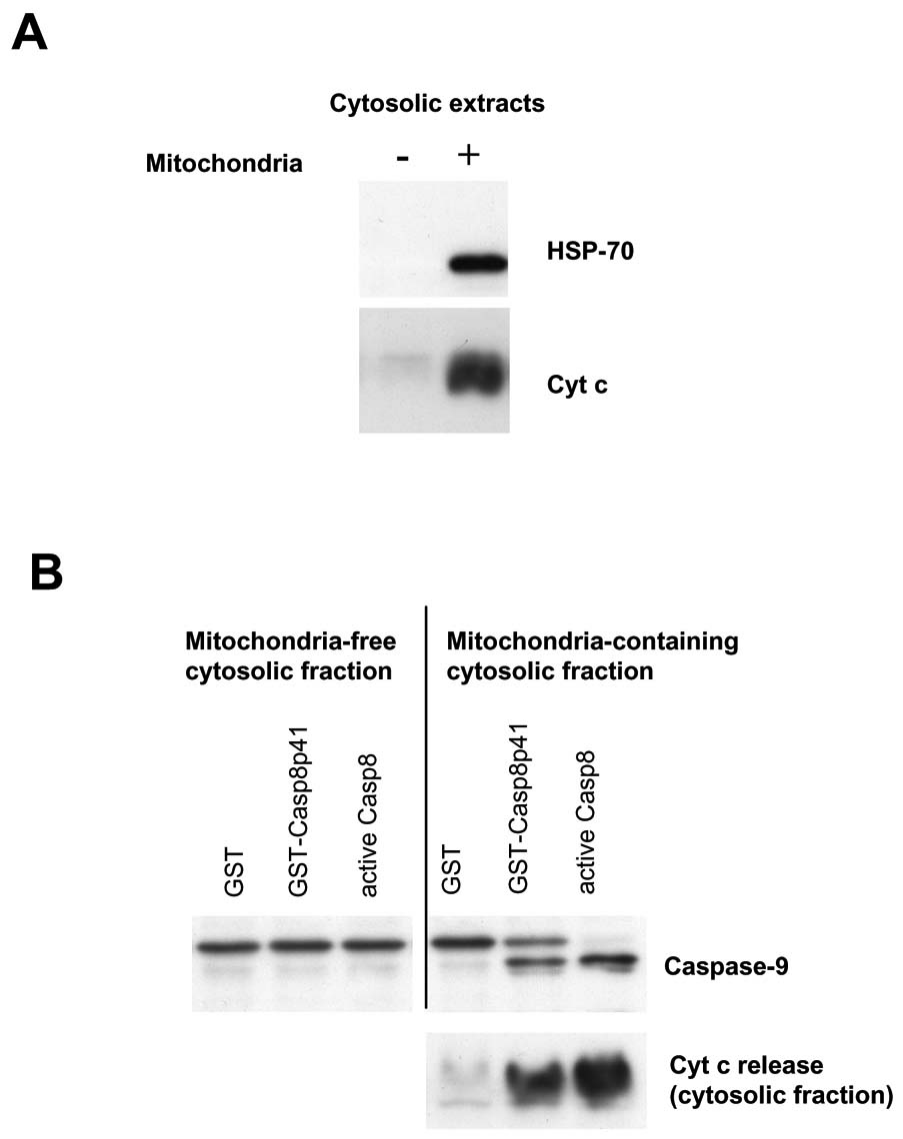
**Casp8p41 acts on mitochondria to release cytochrome c. (A)** Jurkat cells cytosolic extracts containing (+) or lacking (-) mitochondria were analyzed by Western blot for Hsp70 and cytochrome c to confirmed the purity of the fractions. **(B)** Cytosolic extracts containing or lacking mitochondria were incubated with GST alone, GST casp8p41, or with recombinant caspase 8 for 2 hours. Release of cytochrome c into the cytosol and cleavage of caspase 9 were analyzed by Western blot.

We have previously demonstrated that death induced by casp8p41 causes TUNEL positivity, phosphatidylserine externalization, and loss of mitochondrial transmembrane potential [1]. Therefore, it is likely that casp8p41 killing involves mitochondrial depolarization, release of cytochrome c, and downstream activation of caspase 9. This possibility was formally tested using a cell-free system. Cytosols from Jurkat cells were prepared that contained mitochondria or ones that did not. These cytosols were then treated with recombinant GST-casp8p41 or GST alone or with recombinant active caspase 8. Cytosols were blotted for Hsp70 and cytochrome c to ensure purity (Fig. **2A**), and then assessed for caspase 9 cleavage, as well as for cytochrome c release into the cytosolic fraction following treatment. GST treated cytosols had no evidence of caspase 9 processing nor cytochrome c release (Fig. **2B**) Addition of recombinant active caspase 8 resulted in robust cytochrome c release and processing of procaspase 9 into a shorter active form in the mitochondria containing cytosols. GST-casp8p41 treatment caused both caspase 9 processing as well as cytochrome c release, which occurred only in the cytosols that contained mitochondria, but not the cytosols that did not.

### Casp8p41 Acts Directly Upon Mitochondria

**Fig. (3):**
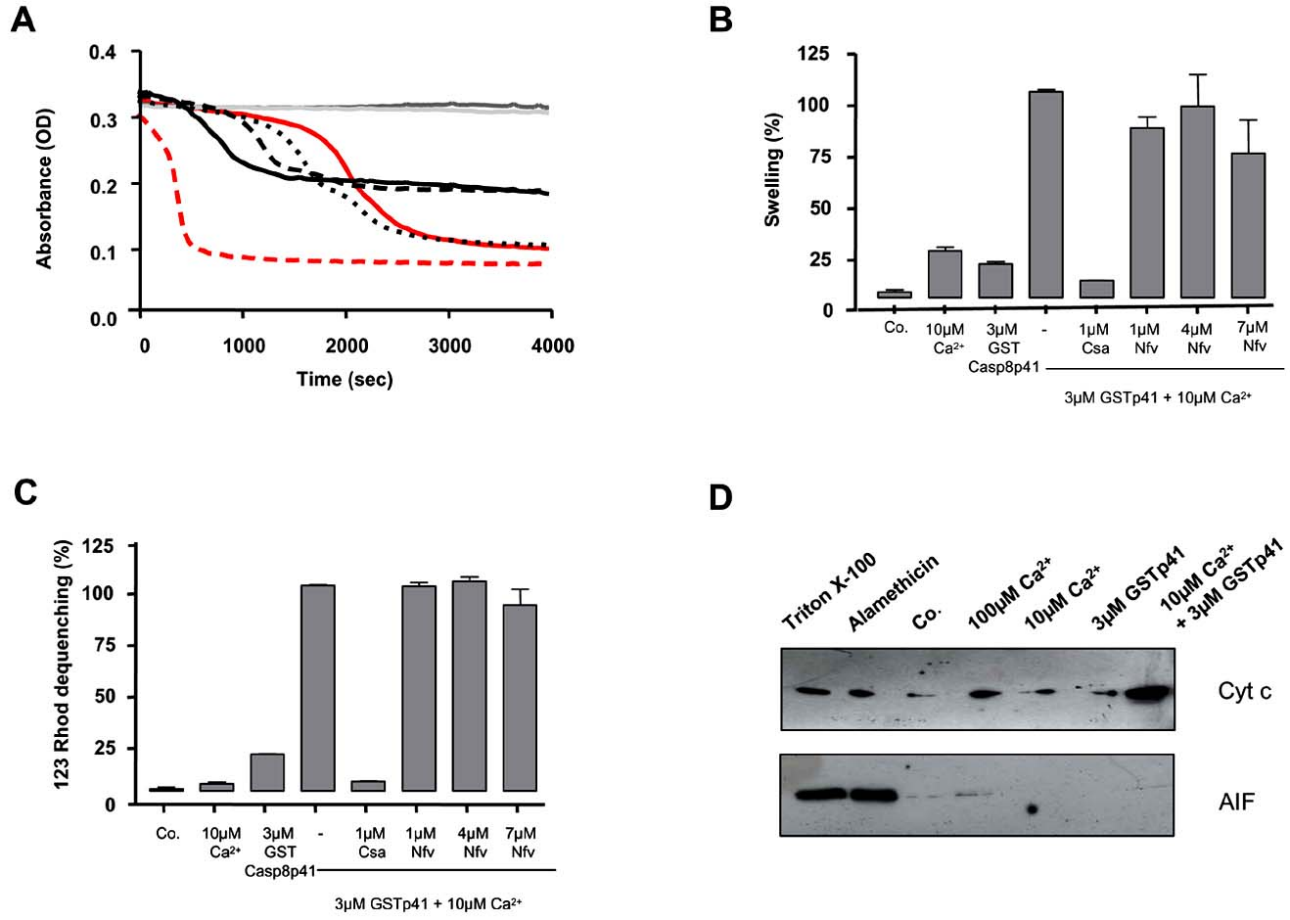
**Mitochondrial effects of casp8p41. (A)** Characterization of casp8p41 induced swelling. Mouse liver isolated mitochondria were suspended in a hypoosmotic buffer, and their absorbance at 540 nm was recorded for 4000 sec at 37°C. Various doses of GST-casp8p41 were added in the presence of 10 µM Ca2+ (red line, 10 µM calcium, small dotted black line, 0.3 µM GST-casp8p41+10 µM calcium, large dotted black line, 1 µM GST-casp8p41 + 10 µM calcium, and black line, 3 µM GST-casp8p41 + 10 µM calcium). 100 µM Ca2+ (red dotted line) serves as a control of maximal swelling. The effect of GST (dark grey line) and GST-casp8p41 (grey line) was evaluated alone at the highest concentration (3 µM). Experiments were repeated three times. **(B)** Various inhibitors (CsA and Nfv) were added 2 min before the inducers, Ca^2+^ and GST-p41, at the indicated doses and their effect evaluated at 17 min. The effect of 3 µM GST-casp8p41 in the presence of 10 µM Ca^2+^ was normalized to 100%. Experiments were repeated three times and error bars represent SD. **(C)** Regulation of GST-casp8p41 induced Δγm loss. The Δγm loss was measured by the 123 Rhodamine dequenching method. Various inhibitors (CsA and Nfv) were added 2 min before the inducers, Ca^2+^ and GST-casp8p41, at the indicated doses. The effect of 3 µM GST-casp8p41 in the presence of 10 µM Ca^2+^ was normalized to 100%. Experiments were repeated three times and error bars represent SD. **(D)** GST-casp8p41 induced the cytochrome c, but not AIF release. Mouse liver isolated mitochondria were incubated in the presence of various agents and the release of cytochrome c, and AIF release was detected in the supernatant of mitochondria by Western-blot. Triton X-100 and alamethicin were used as positive controls of mitochondrial membrane permeabilization and Co. (untreated mitochondria) served as a negative control.

We next questioned whether casp8p41 induces a mitochondrial depolarization directly or indirectly, for example, through a BH3 only Bcl2 family member (such as NOXA, Bid, etc.). Therefore, we tested whether casp8p41 had any effect upon isolated mouse liver mitochondria. Casp8p41 in the presence of 10 µM calcium (Ca^2+^) induced dose-dependent mitochondrial matrix swelling (Fig. **3A**), whose kinetics and amplitude differed from the swelling induced by Ca^2+^, the prototypic ion for permeability transition induction [9] (Fig. **3A**). Ten micromolar of Ca^2+^ is considered as a suboptimal dose, since it did not induce swelling before 1800 sec.

Next, we used a pharmacological approach to analyze the underlying mechanisms. CsA, a potent permeability transition inhibitor, prevents the swelling and the inner membrane depolarization induced by the casp8p41, whereas Nelfinavir (Nfv), an adenine nucleotide transporter (ANT) pore inhibitor [10], promoted a minor inhibition of the swelling and had no inhibitory effect on the inner transmembrane potential (Fig. **3B**,**C**). These results indicate that casp8p41 is able to directly induce permeability transition pore (PTP)-dependent mitochondrial swelling and depolarization, different from that induced by Ca^2+^ and probably independent of ANT. The requirement for suboptimal doses of Ca^2+^ has been previously observed for BH3 mitochondriotoxic peptides derived from Bax and suggests that casp8p41 needs subtle effects of the cation, e.g., on protein conformation, to exert its toxic effect.

The functional consequences of casp8p41-induced mitochondrial alterations were assessed in isolated liver mitochondria treated with casp8p41. In the presence of suboptimal doses of Ca^2+^, but not alone, casp8p41 triggered a strong mitochondrial release of cytochrome c from the intermembrane space (Fig. **3D**), while apoptosis inducing factor (AIF), a pro-apoptotic caspase-dependent effector, was not released.

### Casp8p41 Colocalizes With Mitochondria

**Fig. (4):**
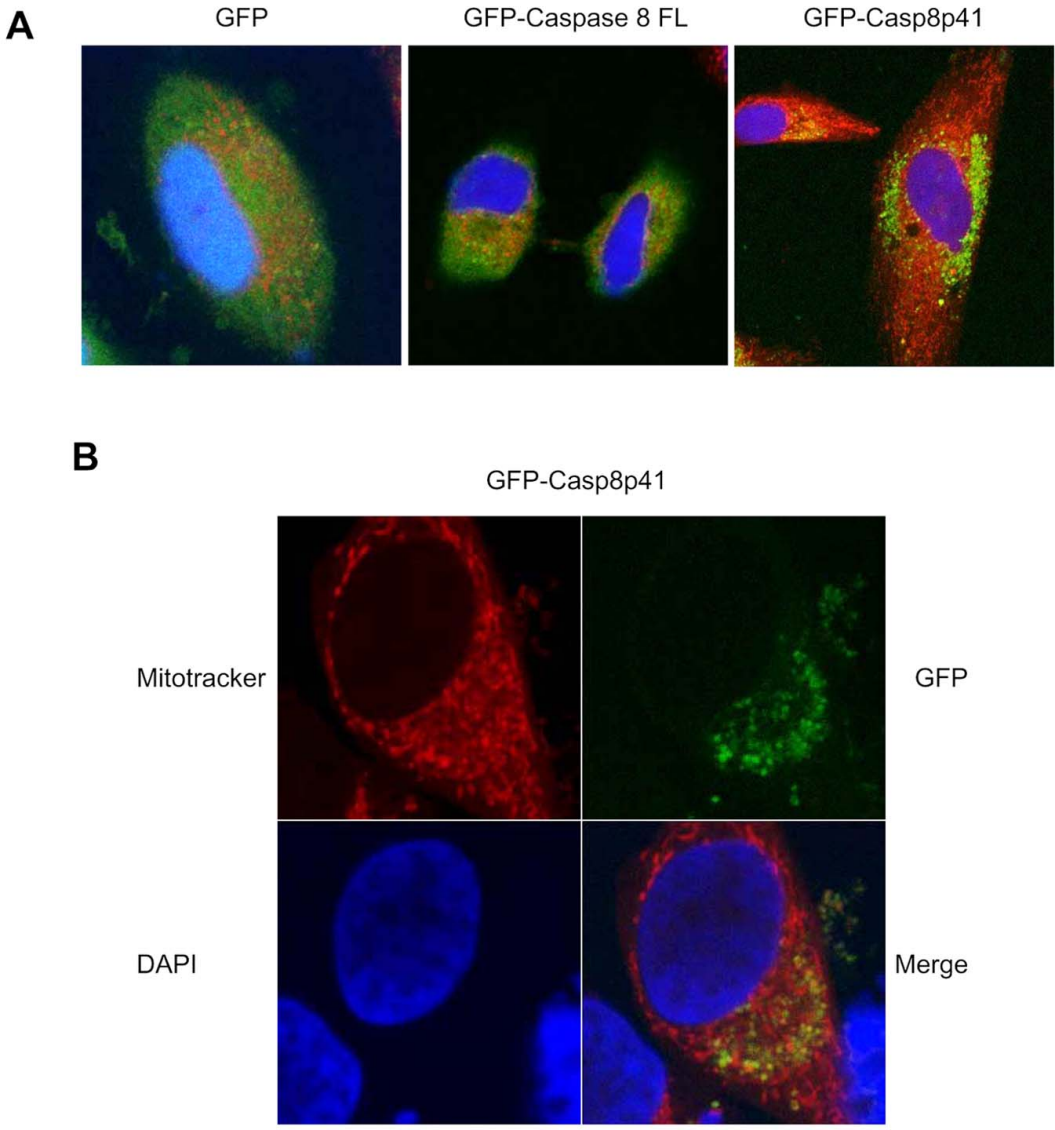
**Casp8p41 colocalizes with mitochondria. (A)** HeLa cells were transfected with GFP, GFP-casp8p41, or GFP-casp8FL. After 6 hours of transfection, cells were stained with MitoTracker^®^ red, and nuclei was counterstained with DAPI. Staining was visualized by confocal microscopy. The figure shows the overlay of the staining: GPF constructs (green), Mitotracker (red), and DAPI (blue). **(B)** Same as in A. Images of GFP-casp8p41 transfected HeLa cells are shown individually for each staining and as an overlay.

Having demonstrated that casp8p41 acts directly upon mitochondria *in vitro*, resulting in mitochondrial depolarization and cytochrome c release, we next assessed the relevance of these events in intact cells. HeLa cells were transfected with GFP alone, GFP full-length procaspase 8, or GFP casp8p41 and stained for mitochondria (red) and nuclear morphology (blue) (Fig. **4A**). GFP control and GFP full-length procaspase 8 transfected cells exhibited diffuse GFP staining with no mitochondrial colocalization. In contrast, GFP casp8p41 transfected cells exhibited punctuate staining (Fig. **4A**) that upon greater magnification colocalized with mitochondria (Fig. **4B**).

## DISCUSSION

Procaspase 8 is a key initiator caspase which is present in the cytosol as an inactive monomer that is critically involved in death receptor-induced apoptosis initiated by Fas, tumor necrosis factor (TNF) receptor 1, or TNF Related Apoptosis Inducing Ligand (TRAIL) receptors 1 or 2 [7]. In such cases, receptor ligation results in recruitment of FADD to the death inducing signaling complex (DISC), whereupon tandem death effector domain (DED) motifs within FADD recruit procaspase 8 *via *homophilic interaction. Such dimerization of caspase 8 permits activation, which then leads to cleavage and production of the mature enzyme comprised of two p10 and two p18 subunits. This model, therefore, requires at least two critical domains within procaspase 8, tandem DEDs which allow homophilic interaction with FADD and the C360 active site which allows catalytic activity. Studies involving FLICE-like inhibitory protein (FLIP) (which contain tandem DEDs with no catalytic site) indicate that catalytically inactive FLIP can form heterodimers with procaspase 8 leading to activation of caspase 8 [11, 12]. Caspase 8 activation can also occur following death receptor-independent stimuli including genotoxic stress [13] or direct cleavage by granzyme B [14] or HIV protease [1, 5]. Having determined that HIV protease cleaves procaspase 8 between phenylalanines 355/356 [1], it was of interest to determine whether and how the cleavage products induced cell death. In theory such cleavage would result in two fragments, one of 41 kd, which contains two DEDs with no active site, and another of 14 kd, which contains an active site but not DEDs.

Autoradiography using S^35^-labeled procaspase 8 resulted in only one p41 kd band (referred to as casp8p41), suggesting that the 14 kd fragment is degraded, and consequently non functional [1]. The combined observations that transfection of casp8p41 into cells rapidly induces cell death and that casp8p41 colocalizes with HIV-infected and apoptotic cells *in vivo* together suggest that casp8p41 itself causes cell death. Given the similarities between FLIP and casp8p41, we postulated a similar mechanism for casp8p41-induced death as for FLIP-induced death. However, casp8p41 pull-down experiments failed to generate caspase 8 activity (data not shown), suggesting that casp8p41 formation of heterodimers with procaspase 8 is not responsible for caspase 8 initiated cell death. This lack of reliance on procaspase 8 was formally tested using the procaspase 8 deficient I9.2 cell line where casp8p41 still efficiently initiated cell death. Our subsequent data demonstrating a direct effect of casp8p41 on mitochondria indicate that caspase 8 cleavage products, including casp8p41, can promote cell death in at least three ways: 1) catalytically through Bid cleavage, 2) formation of heterodimers with procaspase 8 facilitating to induce the proximity model of caspase 8 activation, and 3) the additional pathway of a direct effect on mitochondria causing matrix swelling, loss of transmembrane potential, and cytochrome c release. Based on our pharmacological experiments with isolated mitochondria (Fig. **3**), this effect might involve the induction of the PTP, since the swelling as well as the depolarization induced by casp8p41, can be prevented by cysclosporin A, a prototypic inhibitor of the PTP [15]. As shown by the kinetics analysis and the absence of effect of NFV (Fig. **3**), the permeabilization mechanism is drastically different than from calcium and is independent of the ANT, one putative candidate for the inner membrane pore [16]. A plausible hypothesis would be that casp8p41 interacts with an outer membrane protein such as VDAC, an unknown protein from the inner membrane, and/or with a mitochondrial specific lipid such as cardiolipin, as previously shown for tBid [17]. Domain mapping studies of casp8p41 should provide insights into both the molecular mechanism and mitochondrial target of casp8p41.

## CONCLUSIONS

Although a variety of host and viral factors have been proposed to contributed to T cell death following HIV infection, the relevance of each such mechanism to the overall T cell death seen *in vivo* has been unclear. Two principal reasons contribute to this uncertainty: 1) many studies involve forced overexpression of viral proteins to initiate T cell death, which may or may not replicate the *in vivo* scenario, and 2) that one such cell has become apoptotic. It has been impossible to determine what stimulus was responsible for initiating death signaling. In the case of HIV protease cleavage of procaspase 8, its relevance is suggested by demonstration of casp8p41 within cells from HIV-infected patients. Consequently, enhanced understanding of the molecular mechanisms by which casp8p41 initiates cell death is critical and may lead to novel strategies aimed at inhibiting or at least reducing HIV-induced cell death. The fact that casp8p41 is unique to HIV-infected cell death makes this an appealing therapeutic target. Demonstration that casp8p41 directly causes mitochondrial depolarization provides direction for uncovering the target of casp8p41 and argues that an inhibitor of this process should not interfere with either the DED homophilic interaction nor catalytic functions of caspase 8, which are necessary functions for cellular homeostasis.

## ABBREVIATIONS

TRAIL: = TNF-related apoptosis inducing ligand
HIV: = Human immunodeficiency virus
FADD: = Fas-associated death domain
PTP: = Permeability transition pore
Nfv: = Nelfinavir
AIF: = Apoptosis inducing factor
ANT: = Adenine nucleotide transporter
DED: = Death effector domain
DISC: = Death inducing signaling complex
TNF: = Tumor necrosis factor
FLIP: = FLICE-like inhibitory protein
ATP: = Adenine triphosphate

## References

[1] Nie Z, Bren GD, Vlahakis SR (2007). HIV-1 Protease Cleaves Procaspase 8 *in vivo*. J Virol.

[2] Adams LD, Tomasselli AG, Robbins P, Moss B, Heinrikson RL (1992). HIV-1 protease cleaves actin during acute infection of human T-lymphocytes. AIDS Res Hum Retroviruses.

[3] Shoeman RL, Sachse C, Honer B, Mothes E, Kaufmann M, Traub P (1993). Cleavage of human and mouse cytoskeletal and sarcomeric proteins by human immunodeficiency virus type 1 protease. Actin, desmin, myosin, and tropomyosin. Am J Pathol.

[4] Strack PR, Frey MW, Rizzo CJ (1996). Apoptosis mediated by HIV protease is preceded by cleavage of Bcl-2. Proc Natl Acad Sci USA.

[5] Nie Z, Phenix BN, Lum JJ (2002). HIV-1 protease processes procaspase 8 to cause mitochondrial release of cytochrome c, caspase cleavage and nuclear fragmentation. Cell Death Differ.

[6] Ventoso I, Blanco R, Perales C, Carrasco L (2001). HIV-1 protease cleaves eukaryotic initiation factor 4G and inhibits cap-dependent translation. Proc Natl Acad Sci USA.

[7] Peter ME, Krammer PH (2003). The CD95(APO-1/Fas) DISC and beyond. Cell Death Differ.

[8] Belzacq AS, Vieira HL, Verrier F (2003). Bcl-2 and Bax modulate adenine nucleotide translocase activity. Cancer Res.

[9] Hunter DR, Haworth RA, Southard JH (1976). Relationship between configuration, function, and permeability in calcium-treated mitochondria. J Biol Chem.

[10] Weaver JG, Tarze A, Moffat TC (2005). Inhibition of adenine nucleotide translocator pore function and protection against apoptosis *in vivo* by an HIV protease inhibitor. J Clin Invest.

[11] Chang DW, Xing Z, Pan Y (2002). c-FLIP(L) is a dual function regulator for caspase 8 activation and CD95-mediated apoptosis. Embo J.

[12] Micheau O, Thome M, Schneider P (2002). The long form of FLIP is an activator of caspase 8 at the Fas death-inducing signaling complex. J Biol Chem.

[13] Petak I, Houghton JA (2001). Shared pathways: death receptors and cytotoxic drugs in cancer therapy. Pathol Oncol Res.

[14] Medema JP, Toes RE, Scaffidi C (1997). Cleavage of FLICE (caspase 8) by granzyme B during cytotoxic T lymphocyte-induced apoptosis. Eur J Immunol.

[15] Halestrap AP, Connern CP, Griffiths EJ, Kerr PM (1997). Cyclosporin A binding to mitochondrial cyclophilin inhibits the permeability transition pore and protects hearts from ischaemia/reperfusion injury. Mol Cell Biochem.

[16] Kroemer G, Galluzzi L, Brenner C (2007). Mitochondrial membrane permeabilization in cell death. Physiol Rev.

[17] Gonzalvez F, Pariselli F, Dupaigne P (2005). tBid interaction with cardiolipin primarily orchestrates mitochondrial dysfunctions and subsequently activates Bax and Bak. Cell Death Differ.

